# A nomogram for assessing the risk of deep vein thrombosis following surgery for pilon fractures

**DOI:** 10.1016/j.cjtee.2025.04.006

**Published:** 2026-02-06

**Authors:** Peiyuan Wang, Chengsi Li, Zihang Zhao, Yiran Li, Shuo Yang, Lin Liu, Kuo Zhao, Zhiang Zhang, Lin Jin, Wei Chen, Zhiyong Hou

**Affiliations:** a Orthopaedic Research Institute of Hebei Province, Shijiazhuang, 050051, China; b Department of Orthopaedic Surgery, The Third Hospital of Hebei Medical University, Shijiazhuang, 050051, China; c NHC Key Laboratory of Intelligent Orthopaedic Equipment (The Third Hospital of Hebei Medical University), Shijiazhuang, 050051, China

**Keywords:** Deep vein thrombosis, Pilon fracture, Predictors, Nomogram

## Abstract

**Purpose:**

Deep vein thrombosis (DVT) can occur following fractures of the lower extremities. This study investigated DVT incidence following pilon fractures and analyzed the related risk factors. A nomogram was developed and validated for predicting the likelihood of postoperative DVT.

**Methods:**

From January 2017 to January 2023, data on 1689 patients with pilon fractures at Hebei Medical University's Third Hospital was obtained for this study. The DVT group consisted of DVT cases, while members of the non-DVT group did not have DVT. In order to collect information on demographics, comorbidities, injuries, and laboratory biomarkers, the inpatient medical record system was consulted. The multivariate logistic regression analysis incorporated factors found to be significant (*p* < 0.05) in the univariate analysis. Independent factors predictive of DVT were identified with a backward stepwise regression approach. Predictors were analyzed in R with the construction of a nomogram.

**Results:**

The DVT rate (166 of 1238) in the current study was 13.4%. Using univariate analysis, several DVT predictors were identified, including smoking history (*p* < 0.001), open fracture (*p* = 0.001), fracture blister (*p* < 0.001), body mass index (BMI) (*p* < 0.001), and Elixhauser comorbidity index (ECI) (*p* < 0.001). Furthermore, globulin levels were considerably greater in the DVT group relative to the non-DVT group (*p* = 0.001), while platelets counts were lower (*p* < 0.001). Higher BMI (*p* < 0.001, odds ratio (*OR)* = 0.837, 95% *confidence interval* (*CI)* (0.763 – 0.919)), open fracture (*p* < 0.01, *odd ratio* (*OR)* = 0.431, 95% *CI* (0.286 – 0.649)), fracture blister (*p* = 0.018, *OR* = 0.589, 95% *CI* (0.380 – 0.912)), smoking history (*p* = 0.011, *OR* = 0.561, 95% *CI* (0.360 – 0.874)), and a higher ECI (*p* < 0.001, *OR* = 0.776, 95% *CI* (0.681 – 0.884)) were all strongly associated with DVT, as per the stepwise logistic regression analysis. Five variables independently linked to DVT were the basis for developing the nomogram.

**Conclusions:**

The present research indicates that pilon fractures are more susceptible to DVT than typical ankle fractures, attributed to their high-energy injury characteristics. It was found that BMI, the existence of an open fracture, the lack of blisters, a smoking history, and ECI are independently predictive of DVT following surgery in pilon fracture cases. Consequently, the current study developed a nomogram based on these discovered indicators to facilitate early targeted interventions.

## 1 Introduction

The phrase “pilon fracture”, a type of ankle fracture, is not precisely defined but typically refers to a fracture of the distal third of the tibia, including the articular surface of the distal tibia.^1^ Pilon fractures are relatively common, representing approximately 3% – 10% of tibial fractures.^2^, ^3^, ^4^ Most of these injuries happen around age 45, and males are significantly more likely than women to sustain them.^4^^,^^5^ Furthermore, the fibula is fractured in 75% – 90% of cases.^6^ The severity of the damage is further demonstrated by the generally poor functional outcomes following high-energy pilon fractures. A comprehensive retrospective investigation by Pollak et al.^7^ in 2003 used SF-36 scores obtained from questionnaires on health-related quality of life, finding markedly reduced scores relative to the age-matched control group, and were worsened by chronic comorbidities such as diabetes, asthma, or acquired immune deficiency syndrome. Deep vein thrombosis (DVT) of the lower extremities after major orthopedic surgery is linked with an elevated likelihood of pulmonary embolism and mortality.^8^^,^^9^ Statistical data reveal an annual mortality rate of up to 300,000 individuals in the United States due to pulmonary thromboembolism.^10^ Once major vein thrombosis (such as femoral vein thrombosis or popliteal vein) is diagnosed, patients necessitate intensive and prolonged thrombolytic therapy, alongside repeated ultrasound evaluations. This approach is frequently associated with risks such as bleeding, DVT recurrence, and significant medical costs. Understanding the factors associated with increased DVT risk and implementing preventive measures are essential components of clinical practice. Earlier research has examined the associations between DVT and ankle fractures. While SooHoo et al.^11^ evaluated DVT risk following surgery for ankle fracture, only a limited number of potential factors were incorporated into the multivariate model. Shibuya et al.^12^ used data from the United States National Trauma Data Bank to analyze associations between DVT and pulmonary embolism following ankle trauma; however, they did not specifically address the issue of ankle fractures.

By far, few studies have distinguished pilon fractures from ankle fractures. Due to their unique fracture characteristics, all pilon fractures affect the articular surface to different extents,^1^ presenting difficulties for the construction of models for predicting DVT risk. At the same time, previous studies primarily focused on coagulation indicators. Thus, this study aims to address other potential risk factors, particularly incorporating the patient's preoperative comorbidities index. The aim is to evaluate the possible influence of comorbidities on postoperative thrombosis. To improve the perioperative care of acute trauma, an intuitive nomogram model is created and validated based on these factors to predict the likelihood of postoperative DVT.

## 2 Methods

To successfully carry out the current study, the electronic medical records of all diagnoses and treatments for fractures of the lower extremities at The Third Hospital of Hebei Medical University was undertaken from January 2017 to January 2023. The hospital's institutional review boards granted ethical approval per the 1964 Helsinki Declaration's ethical guidelines. All participants provided their consent for the study.

### 2.1 Study design

**Fig. 1:**
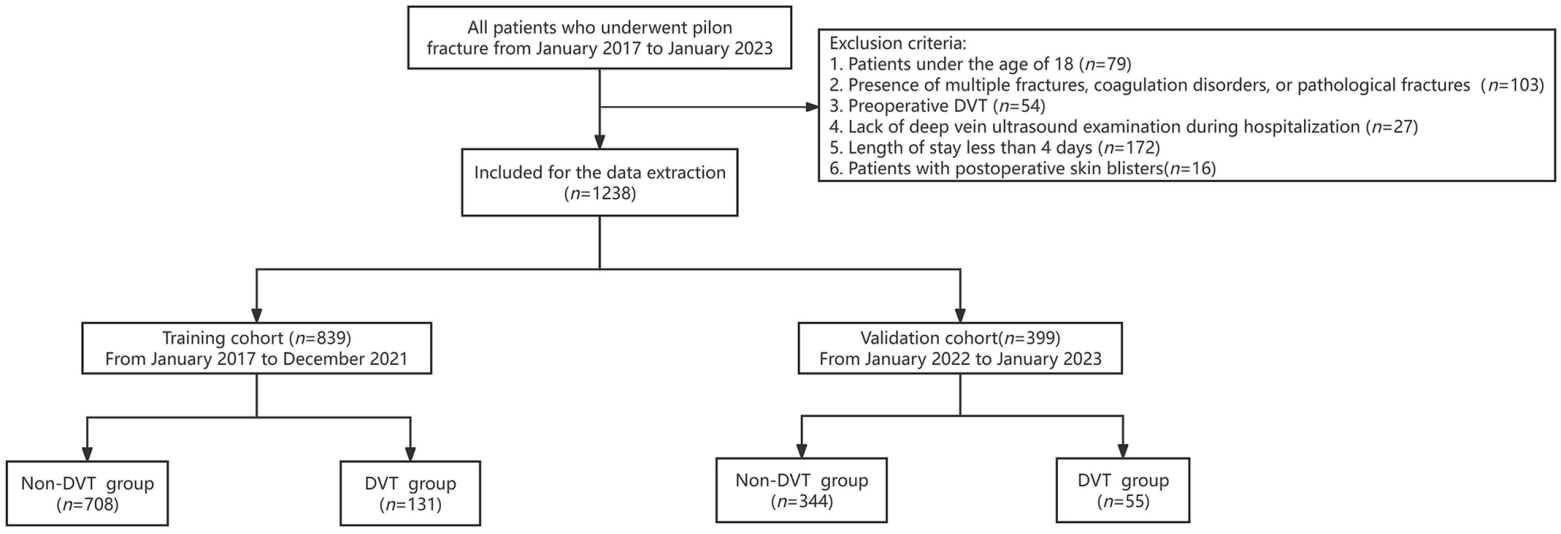
Study flowchart for patient selection.

This retrospective investigation analyzed the data of cases of pilon fracture admitted to our hospital between January 2017 and January 2023. The inclusion criteria were: (1) age ≥18 years; (2) X-ray verification of pilon fracture. The exclusion criteria included: (1) patients <18 years; (2) multiple fractures, coagulation disorders, or pathological fractures; (3) diagnosed with venous thromboembolism before surgery; (4) no deep-vein ultrasound examination while hospitalized; (5) hospitalization <4 days; (6) patients exhibiting postoperative skin blisters (after the operation, there were local transparent blisters in the wound, which were considered to be edema of the wound rather than tension blisters generated after fracture) (Fig. 1).

Demographic, clinical, and laboratory information were rigorously gathered for this investigation. Fracture types, body mass index (BMI), gender, age, fracture blisters, Elixhauser comorbidity index (ECI) (Table 1), history of alcohol and smoking, intake of a dehydrating agent, interval between injury and admission, and injury mechanism were among the demographic characteristics.

**Table 1:** Elixhauser comorbidity index.

Complication	Score
Metastatic malignancy	12
Liver disease	11
Lymphoma	9
Congestive heart failure, paralysis	7
Neurodegenerative disease, weight loss	6
Arrhythmias, disturbance of water and electrolyte balance, and renal failure	5
Unmetastasized solid tumors, pulmonary circulatory disease	4
Chronic pulmonary disease, coagulation dysfunction	3
Peripheral vascular disease	2
Hypertension, diabetes with/without complications, hypothyroidism, peptic ulcer without bleeding, HIV/AIDS infection, rheumatoid arthritis/collagenous vascular disease, alcoholism, mental illness	0
Valvular heart disease	-1
Hemorrhagic anemia, nutritional deficiency anemia	-2
Depression	-3
Obesity	-4
Drug abuse	-7

HIV: human immunodeficiency virus; AIDS: acquired immune deficiency syndrome.

A comprehensive investigation was performed on various postperative laboratory parameters, such as white blood cells, red blood cells, platelets, neutrophils, mean platelet volume, monocytes, mean corpuscular hemoglobin concentration, lymphocytes, hemoglobin, hematocrit, eosinophils, basophils, uric acid, urea, total CO_2_, total cholesterol, triglyceride, lactic dehydrogenase, glucose, direct bilirubin, creatinine, creatine kinase, cholinesterase, globulin, Cl, P, Mg, Na, K, Ca, alanine transaminase, aspartate aminotransferase, alkaline phosphatase, and albumin.

### 2.2 Diagnostic methods and standards of DVT

Doppler ultrasonography (DUS) was used as the diagnostic method to confirm the DVT existence. Vessel incompressibility, lumen blockage or filling defects, lack of respiratory motion in the venous segment above the knee, and inadequate increase in flow to the calf or foot following compression were all part of the diagnostic criteria for DVT. The range of scanning covered deep veins in the lower parts of the legs from the inguinal ligament to the ankle. However, intramuscular veins, such as those in the gastrocnemius, were not included due to their poor clinical significance.

In our hospital, trauma patients routinely undergo DUS examinations within 24 h of admission, followed by subsequent assessments every 3 to 7 days while awaiting surgical intervention. The participants were advised to raise the injured extremity from arrival following our institution's recognized treatment regimen for lower limb fractures. Therapeutic dosages of anticoagulant medications, such as enoxaparin sodium injection at a dosage of 100 Anti-Xa IU/kg, are administered twice daily to patients with positive DUS results. Furthermore, preventative doses of blood-thinning medications, such as an injection of enoxaparin sodium at a dosage of 4000 Anti-Xa IU once a day, are given to people who are at a higher risk of thrombosis, particularly the elderly. In cases where patients face prolonged waiting periods for surgery, it is customary to advise increased water intake and appropriate limb movements. Furthermore, intermittent pneumatic pressure pumps facilitate blood circulation in the lower extremities, reducing the risk of thrombosis.

### 2.3 Statistical analysis

SPSS 25.0 (IBM Corp., NY, USA) was used for data analysis, with *p* < 0.05 considered statistically significant. Data distributions were examined using Shapiro-Wilk tests. Continuous variables are shown as mean ± standard deviation (SD), with *t*-tests used for analysis of normally distributed data and Mann-Whitney *U* tests for non-normally distributed data. *Chi*-square and Fisher's exact tests were used for comparing categorical variables, which are shown as percentages and numbers.

The multivariate logistic regression analysis incorporated variables that had shown significance (*p* < 0.05) in the univariate analysis. Backward stepwise regression was utilized for risk factor identification. The selected predictors were analyzed further in R (version 3.6.5), and the “rms” package was utilized for nomogram construction.

Several assessment criteria, such as decision curve analysis (DCA), calibration curves, receiver operating characteristic (ROC) curves, and the conformance index (C-index), were used to evaluate the model's predictive capability and application value. The area under the ROC curve (AUC) and the C-index, which varied from 0.5 to 1.0, showed positive associations with discriminant capacity and prediction accuracy of the nomogram.

The concordance between diagnosed and predicted thrombosis was evaluated using the calibration curve. When the predicted and ideal curves are more aligned, a larger correlation exists between the expected and actual probability. The nomogram model's clinical utility was assessed using DCA, which considers threshold probabilities and net benefit. The improved C-index was calculated using 1000 sample iterations, and the Bootstrap approach was used for internal verification to validate the model's prediction ability further. Next, the present study compared the nomogram's DCA, calibration, and ROC curves in the training and validation sets using the validation set for additional external verification. For every analysis, a significance level of 0.05 was used.

## 3 Results

A total of 451 of the 1689 cases that were assessed were not included in the research. There were 1238 patients in this study, 1099 of whom were men and 139 of whom were women, and all had had surgery before. With 166 patients affected and 1072 unaffected, the postoperative incidence of DVT was 13.4%. As shown in Table 2, there are 399 instances in the validation set and 839 cases in the training set. The DVT and non-DVT groups differed significantly in terms of smoking history, ECI, time from injury to admission (*p* < 0.001), open fracture (*p* = 0.001), and BMI (*p* < 0.001), according to the statistical analysis. In particular, BMI values were greater in the DVT group relative to non-DVT cases. Further, individuals in the DVT group exhibited a higher prevalence of smoking history and open fractures relative to those in the non-DVT group. Furthermore, higher ECI was associated with greater DVT risk. No differences were seen in terms of age, sex, fracture blister presence, mechanism of injury, drinking, or use of dehydrating medications (*p* > 0.05).

**Table 2:** Overview of patient data about DVT.

Variable	Non-DVT group (*n* = 708)	DVT group (*n* = 131)	*p* value
Age (year), median (Q_1_, Q_3_)	43.00 (28.75, 59.00)	42.00 (31.00, 58.50)	0.939
Gender, *n* (%)			0.808
Female	73 (10.3)	12 (9.2)	
Male	635 (89.7)	119 (90.8)	
BMI (kg/m^2^), median (Q_1_, Q_3_)	23.90 (21.30, 25.80)	25.70 (23.80, 27.10)	<0.001
Open fracture, *n* (%)			0.001
No	455 (64.3)	64 (48.9)	
Yes	253 (35.7)	67 (51.1)	
Mechanism of injury, *n* (%)			0.793
Flat land tumble	211 (29.8)	36 (27.5)	
Traffic injury	243 (34.3)	44 (33.6)	
High falling	254 (35.9)	51 (38.9)	
Time from injury to admission (days), *n* (%)			<0.001
<1	466 (65.8)	59 (45.0)	
1 – 6	138 (19.5)	29 (22.1)	
≥7	104 (14.7)	43 (32.8)	
Alcohol history, *n* (%)			0.268
No	211 (29.8)	46 (35.1)	
Yes	497 (70.2)	85 (64.9)	
Smoking history, *n* (%)			<0.001
No	539 (76.1)	67 (51.1)	
Yes	169 (23.9)	64 (48.9)	
Dehydrating agent, *n* (%)			0.307
No	266 (37.6)	56 (42.7)	
Yes	442 (62.4)	75 (57.3)	
Fracture blister, *n* (%)			1.000
No	248 (35.0)	46 (35.1)	
Yes	460 (65.0)	85 (64.9)	
ECI, median (Q_1_, Q_3_)	4.00 (2.00, 4.00)	4.00 (3.00, 7.00)	<0.001

DVT: deep vein thrombosis; BMI: body mass index; ECI: Elixhauser comorbidity index.

The laboratory test results are shown in Table 3. Globulin levels were considerably greater in DVT compared with non-DVT cases (*p* = 0.001), while platelet counts were lower (*p* < 0.001). Other laboratory data, however, showed no discernible changes between the groups (all *p* > 0.05).

**Table 3:** Patient laboratory outcomes, median (Q_1_, Q_3_).

Variable	Non-DVT group (*n* = 708)	DVT group (*n* = 131)	*p value*
Basophil (10^9^/L)	0.04 (0.02, 0.05)	0.04 (0.02, 0.05)	0.677
Eosinophil (10^9^/L)	0.14 (0.07, 0.16)	0.14 (0.07, 0.16)	0.797
Hematocrit (%)	36.20 (34.00, 40.39)	36.22 (34.29, 38.56)	0.534
Hemoglobin (g/L)	123.08 (113.22, 136.49)	123.08 (114.67, 131.90)	0.528
Lymphocyte (10^9^/L)	1.64 (1.23, 1.77)	1.59 (1.23, 1.72)	0.290
Monocyte (10^9^/L)	1.06 (0.73, 1.07)	1.06 (0.80, 1.09)	0.152
Mean platelet volume (fL)	8.87 (8.18, 9.12)	8.91 (8.23, 9.27)	0.219
Neutrophil (10^9^/L)	12.60 (8.10, 16.10)	11.50 (7.70, 14.60)	0.013
Creatine kinase (MB form) (U/L)	101.01 (38.98, 157.70)	97.17 (37.00, 144.65)	0.097
Platelet (10^9^/L)	226.94 (182.18, 251.68)	200.35 (174.77, 239.12)	<0.001
Red blood cell (10^12^/L)	4.63 (3.68, 7.89)	4.82 (3.68, 7.62)	0.893
White blood Cell (10^9^/L)	15.60 (11.92, 16.80)	15.92 (12.10, 16.92)	0.069
Albumin (g/L)	34.35 (33.03, 39.23)	35.09 (33.16, 36.48)	0.447
Alkaline phosphatase (U/L)	72.09 (56.98, 73.77)	70.87 (63.34, 73.33)	0.373
Alanine transaminase (U/L)	50.29 (28.82, 61.06)	51.29 (30.65, 54.72)	0.615
Aspartate aminotransferase (U/L)	68.62 (31.93, 140.92)	92.22 (34.45, 107.31)	0.947
Ca (mmol/L)	2.06 (1.98, 2.20)	2.09 (2.00, 2.18)	0.748
Cholinesterase (kU/L)	6.40 (5.57, 6.82)	6.57 (5.76, 6.84)	0.059
Creatine kinase (U/L)	1991.79 (420.62, 5184.29)	2717.11 (522.72, 3870.34)	0.994
CL (mmol/L)	104.43 (102.81, 105.89)	104.42 (102.63, 105.33)	0.066
Creatinine (μmol/L)	72.63 (62.07, 73.63)	71.07 (66.70, 73.60)	0.564
Direct bilirubin (μmol/L)	5.29 (3.41, 6.02)	5.35 (4.28, 5.79)	0.893
Globulin (g/L)	21.88 (19.56, 22.56)	22.05 (20.83, 22.79)	0.001
Glucose (mmol/L)	7.95 (6.41, 8.08)	7.96 (6.77, 8.17)	0.196
K (mmol/L)	4.03 (3.68, 4.16)	4.03 (3.76, 4.13)	0.671
Lactic dehydrogenase (U/L)	758.88 (276.62, 908.19)	752.62 (334.41, 815.11)	0.480
Mg (mmol/L)	0.83 (0.77, 0.94)	0.82 (0.78, 0.92)	0.672
Na (mmol/L)	137.36 (136.01, 139.31)	138.13 (135.88, 139.00)	0.669
P (mmol/L)	1.18 (0.99, 1.24)	1.17 (1.05, 1.23)	0.933
Total cholesterol (mmol/L)	3.26 (2.80, 3.69)	3.31 (2.92, 3.46)	0.341
Total CO_2_ (mmol/L)	23.52 (22.09, 25.06)	23.44 (22.36, 24.53)	0.439
Triglyceride (mmol/L)	1.38 (0.84, 1.39)	1.38 (1.00, 1.39)	0.572
Total protein (g/L)	56.66 (52.66, 62.50)	57.17 (53.80, 62.42)	0.191
Uric acid (μmol/L)	329.01 (266.09, 356.14)	325.86 (282.25, 344.39)	0.309
Ureophil (mmol/L)	5.69 (4.53, 5.80)	5.50 (4.65, 5.78)	0.269

The logistic regression analysis revealed that the DVT was closely linked with higher BMI (*p* < 0.001, *odds ratio* (*OR)* = 0.837, 95% *confidence interval* (*CI)* (0.763 – 0.919)), an open fracture (*p* < 0.001, *OR* = 0.431, 95% *CI* (0.286 – 0.649)), a fracture blister (*p* = 0.018, *OR* = 0.589, 95% *CI* (0.380 – 0.912)), a smoking history (*p* = 0.011, *OR* = 0.561, 95% *CI* (0.360 – 0.874)), and a higher ECI (*p* < 0.001, *OR* = 0.776, 95% *CI* (0.681 – 0.884), *p* < 0.001). In addition, as shown in Table 4, this study did not find any other factors impacting the incidence of DVT in these patients.

**Table 4:** Binary logistic regression study of DVT-related factors.

Characteristics	*OR*	95% *CI*	*p* value
BMI	0.837	0.763 – 0.919	<0.001
Open fracture	0.431	0.286 – 0.649	<0.001
Fracture blister	0.589	0.380 – 0.912	0.018
Smoking history	0.561	0.360 – 0.874	0.011
ECI	0.776	0.681 – 0.884	<0.001

DVT: deep vein thrombosis; *OR*: odds ratio; *CI*: confidence interval; BMI: body mass index; ECI: Elixhauser comorbidity index.

**Fig. 2:**
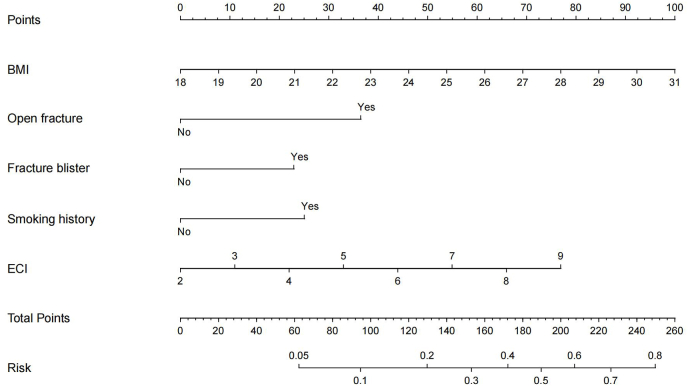
The nomogram employed to forecast DVT risk before surgery. BMI: body mass index; ECI: Elixhauser comorbidity index; DVT: deep vein thrombosis.

**Fig. 3:**
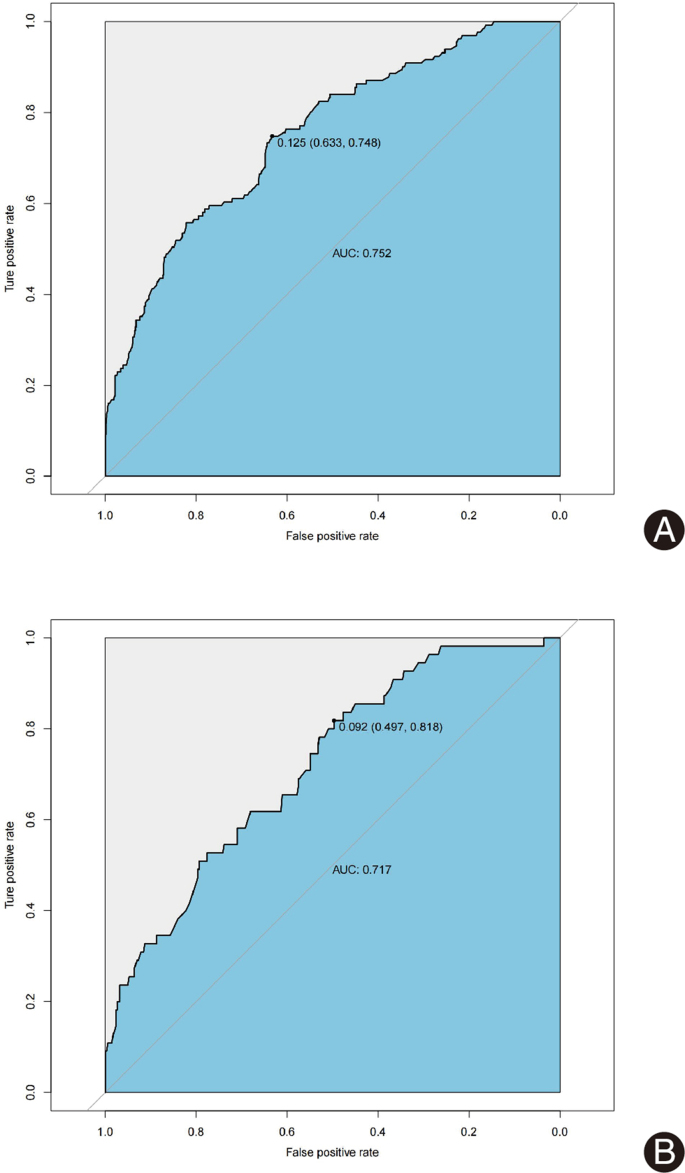
The nomogram's ROC curves for the training (A) and validation (B) sets. The AUC positively correlated with the nomogram's predictive accuracy. The model had high discriminative performance, as seen by the training and validation sets' respective AUCs of 0.752 and 0.717 for the nomogram. ROC: receiver operating characteristic; AUC: area under the ROC curve.

**Fig. 4:**
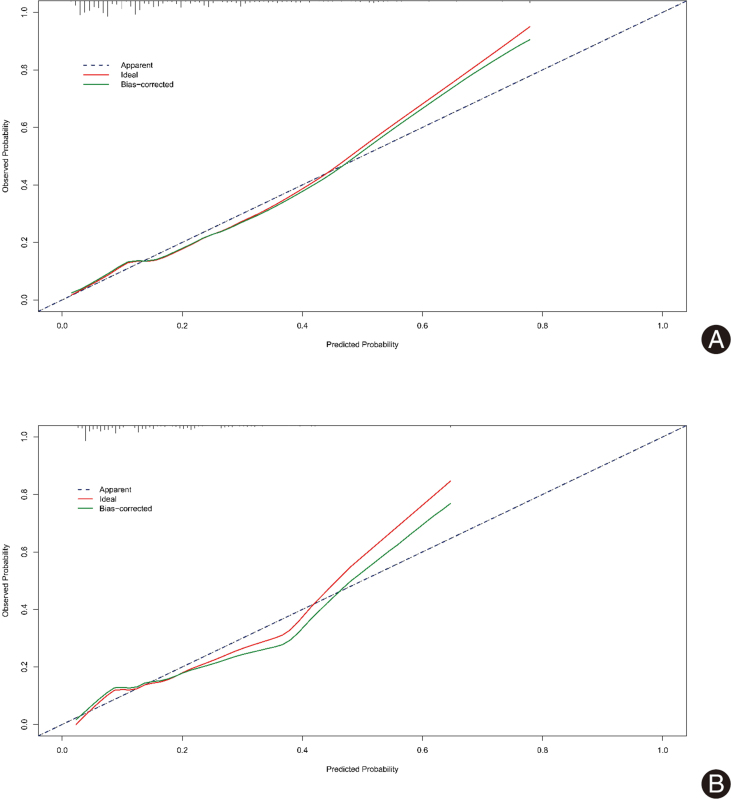
Nomogram calibration curves for the training (A) and validation (B) sets. The model's anticipated probability is shown on the X-axis, while the actual probability is shown on the Y-axis. The predictive consistency of the nomogram improves as the distance between the red and green curves and the ideal dashed line increases.

**Fig. 5:**
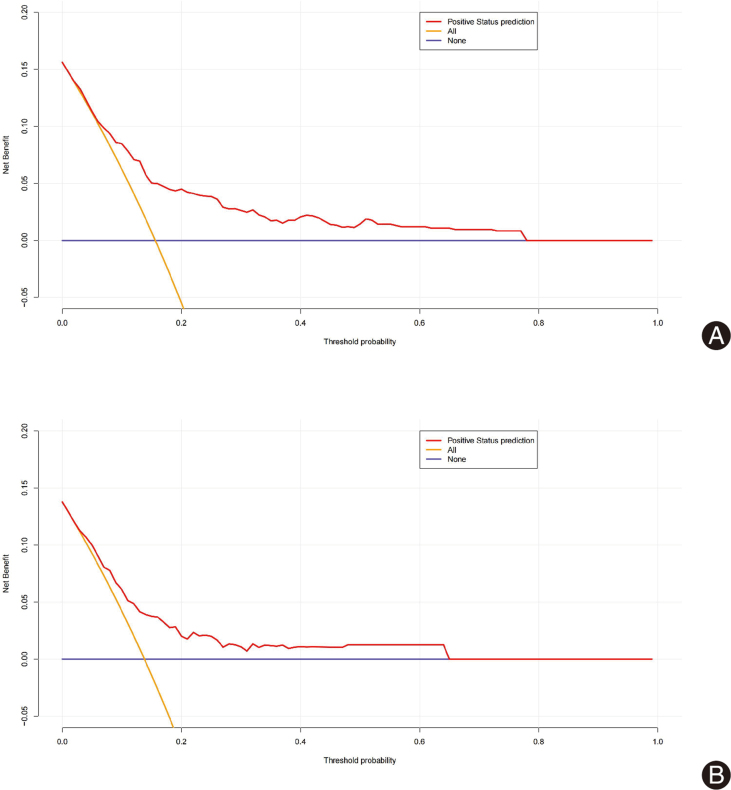
The nomogram's DCA for the training and validation sets (A and B). DCA shows that the training model's net benefit is more significant in the 5% – 78% threshold probability interval, while the validation model's net benefit is larger in the 6% – 64% threshold probability region. DCA: decision curve analysis.

A nomogram intended to forecast the probability of postoperative DVT was created using the results collected (Fig. 2). The performance and dependability of the nomogram were then thoroughly assessed in the current study. With a sensitivity of 74.8% and a specificity of 63.3%, the prediction model's AUC showed a strong discriminative capacity at 0.752 (95% *CI* (0.707 − 0.798)) (Fig. 3). These indicators highlight how well the model predicts risk. With a modified value of 0.744 after 1000 bootstrap verifications, the nomogram's C-index was 0.752, demonstrating impressive model refinement. Further, the consistency between the nomogram's predicted and actual probabilities of DVT is shown by the calibration curve (Fig. 4). In addition, in comparison to no intervention, the DCA of the nomogram demonstrated a significant net benefit, especially within the 5% – 78% threshold probability range (Fig. 5). This shows, even more, how valuable the DVT prediction nomogram is for clinical decision-making.

A nomogram that included 5 variables was created, as shown in Fig. 1. The subject's BMI (kg/m^2^) is first found on the relevant variable axis to properly use the nomogram. The equivalent score is obtained by drawing a vertical line intersecting the “Points” axis. This procedure is repeated for every covariate; the scores are added over time. The likelihood of acquiring DVT is then calculated by matching the cumulative score with the relevant point on the “Total Points” axis and drawing a vertical line that intersects the “Risk of DVT” axis.

## 4 Discussion

This is the first nomogram constructed to predict the likelihood of DVT in individuals with single pilon fractures. Multifactorial analysis revealed independent risk variables for thrombosis, including BMI, open fracture, fracture blister, smoking history, and ECI, and the results show a 13.4% incidence of DVT. The nomogram, constructed from these 5 factors, confirmed strong performance metrics, with an AUC value of 0.752 (95% *CI* (0.707 – 0.798)), a sensitivity of 74.8%, and a specificity of 63.3%. The model was validated internally and externally, demonstrating consistency.

Unlike normal ankle fractures, pilon fractures tend to be caused by high-energy trauma accompanied by marked axial force, causing fracture of the tibial plafond over the talus.^13^ A retrospective study involving 1451 patients with ankle fractures conducted by Zixuan et al.^14^ found a DVT incidence of 2.6%, significantly lower than the 13.4% incidence observed in pilon fracture patients in this study. This may result from high energy damage, which increases the likelihood of DVT, aligning with the findings of Park et al.^15^ regarding hip fractures associated with high energy. Consistent with this study, Basques et al.^16^ observed that heart disease, obesity, and dependent functional status were independently linked to thromboembolic outcomes following ankle fracture procedures. Specifically, by substituting BMI for obesity and optimizing comorbidities for ECI, this study evaluates the risk factors for DVT in a tangible and quantifiable manner.

Numerous studies have proven a clear link between BMI and the incidence of DVT following surgical procedures.^17^, ^18^, ^19^ The causal link between BMI and DVT can be attributed to diverse biological mechanisms. Primarily, mechanical factors like venous stasis are likely to impact DVT progression. High BMI's influence on venous return has been documented, potentially increasing susceptibility to chronic venous insufficiency and DVT.^20^ Additionally, inflammation, including chronic low-grade inflammation and inflammatory factors such as C-reactive protein and albumin, contributes markedly to the causal link between raised BMI and DVT.^21^^,^^22^ Elevations in plasminogen activator inhibitor-1 in the blood have also been linked to a higher risk of defective fibrinolysis, which prevents blood clots from dissolving.^23^ Notably, the observed increase in venous thromboembolism diagnoses^24^ aligns with the prevalence of high BMI,^25^ further substantiating the direct link between high BMI and DVT. Existing guidelines identify a BMI ≥ 30 kg/m^2^ as a significant determinant of DVT. The World Health Organization employs a BMI ≥ 30 kg/m^2^ as the standard for obesity,^26^ while the World Health Organization Western Pacific Region and the International Working Group recommend a BMI > 25 kg/m^2^ as the obesity threshold for the Asian population.^27^ On the contrary, the Chinese Obesity Working Group contends that a BMI ≥ 28 kg/m^2^ is more suitable for defining obesity.^28^ The findings of this study underscore high BMI as a risk factor influencing the development of DVT. Consequently, heightened vigilance for DVT is warranted in patients with a high level of BMI.

During neutrophil phagocytosis of pathogens, the phagosome accumulates antibacterial factors that release a fibrous network consisting of chromatin and granular protein. This network, recognized as neutrophil extracellular traps (NETs), assumes a critical role in the degradation of toxic factors and the eradication of bacteria.^29^ Liu et al.^30^ have observed a significant involvement of NETs in the thrombotic processes among these patients. Other related studies have also provided evidence of NETs adhering to platelets, activating factor XII, and stimulating the production of thrombin, thus promoting thrombosis.^31^^,^^32^ Abnormal coagulation factors in Globulin (such as coagulation factors Ⅷ, Ⅸ, Ⅹ, etc.) may lead to blood hypercoagulability, while abnormal inflammatory response and immunoglobulin function may further destroy vascular endothelium and release pro-coagulant substances, thus increasing the risk of DVT.^33^ At the same time, abnormal quantity and function of platelets, such as increased adhesion and aggregation ability, may exacerbate platelet aggregation at vascular endothelial injury, and interaction with substances such as von Willeophilia factor may also promote thrombosis.^34^ Notably, patients with open fractures are particularly susceptible to bacterial invasion, which elevates the bloodstream's neutrophil count, inducing a hypercoagulable state in the body.

Fracture blisters, occurring relatively infrequently in high-energy fractures, have a reported incidence of 2.9%. However, in tibial fractures, it has been noted that a clear fluid or blood-filled blister can emerge as early as 6 h post-fracture. According to Guo et al.,^35^ these blisters are an efficient pressure-relieving mechanism in addition to causing skin damage. Concurrently, blister formation is one way to reduce abnormally high pressure inside a compartment, according to Varela et al.^36^ poor blood circulation is a significant contributor to DVT. When pilon fractures occur, the fracture blisters indicate increased pressure in the fascial compartment, poor blood circulation, and a higher risk of developing DVT.

Results presented by other scholars have previously indicated that smoking elevates blood coagulation factors, including plasma fibrinogen, initiating the intrinsic coagulation pathway. This process impairs fibrinolysis, damages the endothelial wall, and activates the inflammatory system.^37^ This emphasizes the crucial need for the prompt secretion of tissue plasminogen activator from vascular endothelia to ensure effective fibrinolysis. However, research has indicated compromised acute tissue plasminogen activator release in the endothelium of individuals who smoke cigarettes.^38^ Consequently, individuals who smoke are more susceptible to developing DVT. Furthermore, an observed association exists between a higher burden of comorbidity and the occurrence of DVT. Other related studies have demonstrated that an increased burden of comorbidity in various orthopedic conditions significantly raises the risk of severe adverse events, including venous thromboembolism.^39^^,^^40^

This study offers notable scientific insights, including formulating a nomogram and its thorough validation through internal and external assessments. Nevertheless, it is crucial to recognize several limitations. Firstly, the inevitability of selection bias is an inherent drawback stemming from the study's retrospective nature. Second, some factors, such as the length of limb immobilization, that might affect the incidence of DVT were either not measured or were not documented. Third, data were obtained from a single center, thus potentially restricting the generalizability of the findings. Therefore, to demonstrate the strong clinical value of the suggested approach, prospective observational studies incorporating multicenter data are crucial.

Nomograms, frequently used in clinical predictive model studies,^41^, ^42^, ^43^, ^44^ use each risk factor's predictive value to represent the final predictions visually. In order to help doctors determine the likelihood of postoperative DVT in newly admitted cases of pilon fractures, the current study has effectively created and validated a nomogram. Five predictors from routine tests performed shortly after admission are incorporated into the nomogram. The nomogram's application speeds up the process of identifying people who are more likely to develop DVT. Clinicians can assess the prediction likelihood for specific variables using the vertical line corresponding to the results for that variable and adding them up to determine the corresponding risk value. To identify the presence of DVT and prevent its progression, it is imperative to promptly conduct ultrasound examinations for patients deemed at elevated risk, accompanied by aggressive pharmacological intervention. Timely intervention in patients at high risk of thrombosis requires a combination of measures, including basic prevention (such as early activity and avoidance of dehydration), pharmacoprophylaxis (such as the use of anticoagulants and adjusting the dose to the risk of bleeding), mechanical prevention (such as the use of intermittent pressurized inflators), and monitoring and follow-up (regularly assessing the risk of thrombosis and monitoring for adverse drug reactions).

Limitations of this study merit consideration. First, its single-centre, retrospective design inherently invites selection bias and limits external validity; unmeasured confounders (e.g., limb immobilization duration, outpatient anticoagulant compliance) could not be captured. Second, DVT screening was performed at the discretion of the treating team rather than by a rigid protocol, so subclinical events may have been missed. Third, the nomogram was derived from a Chinese cohort of trauma patients, which may restrict generalizability to other ethnicities, sex distributions, or elective orthopaedic populations. Fourth, we did not externally validate the model in prospective multicentre data; internal bootstrap validation may over-estimate performance. Finally, the study spans 6 years during which anticoagulation practices evolved; temporal trends were not formally analyzed. Prospective, multicentre validation with standardised ultrasound screening and complete medication records is warranted before clinical deployment.

In summary, the current study has shown that pilon fractures are more likely to result in DVT than typical ankle fractures due to their high-energy injury characteristics and many critical factors that are crucial predictors of the likelihood of developing DVT after surgery in cases of isolated pilon fractures. BMI, an open fracture, blisters, a history of smoking, and the ECI are all included in these considerations. To enhance the precision of risk assessment, this study has meticulously devised a nomogram grounded in these identified predictors. The nomogram has demonstrated excellent discriminating ability and usefulness in clinical settings after being verified internally and externally. The ongoing evaluation of the model's performance via validation methods has produced consistently positive results. This nomogram serves as a valuable tool for healthcare practitioners, facilitating the rapid identification of postoperative DVT risk in hospitalized cases of pilon fractures. This reduces the prompt execution of interventions, thereby enhancing patient care outcomes.

## CRediT authorship contribution statement

**Peiyuan Wang:** Writing – review & editing, Visualization, Writing – original draft, Validation. **Chengsi Li:** Writing – original draft, Writing – review & editing, Visualization. **Zihang Zhao:** Formal analysis, Data curation. **Yiran Li:** Writing – review & editing, Visualization, Writing – original draft. **Shuo Yang:** Writing – review & editing, Writing – original draft. **Lin Liu:** Validation, Supervision. **Kuo Zhao:** Writing – original draft, Writing – review & editing, Visualization. **Zhiang Zhang:** Writing – review & editing, Visualization, Writing – original draft. **Lin Jin:** Writing – original draft, Writing – review & editing, Visualization. **Wei Chen:** Supervision. **Zhiyong Hou:** Supervision.

## Ethical statement

This retrospective study was approved by the Institutional Review Board of the 3rd Hospital of Hebei Medical University (K2023-107-1) before collecting data.

## Funding

The research was supported by Key R&D Program of the China Ministry of Science and Technology (2024YFC2510600); Hebei Province National Guidance for Local Science and 10.13039/100006180Technology Development Fund Project (246Z7701G); and National-Level Scientific Research Innovation Team Cultivation Project of 10.13039/501100004880Xinjiang Medical University (20232111235); the 10.13039/501100001809National Natural Science Foundation of China (82072523); the funder had no role in study design, data collection and analysis, decision to publish, or preparation of the manuscript.

## Declaration of competing interest

There are no competing interests.

## References

[1] Bone L.B. (1987). Fractures of the tibial plafond. The pilon fracture. Orthop Clin N Am.

[2] Luo T.D., Eady J.M., Aneja A. (2017). Classifications in brief: Rüedi-Allgöwer classification of tibial plafond fractures. Clin Orthop Relat Res.

[3] Chen H., Cui X., Ma B. (2019). Staged procedure protocol based on the four-column concept in the treatment of AO/OTA type 43-C3.3 pilon fractures. J Int Med Res.

[4] Mauffrey C., Vasario G., Battiston B. (2011). Tibial pilon fractures: a review of incidence, diagnosis, treatment, and complications. Acta Orthop Belg.

[5] Cutillas-Ybarra M.B., Lizaur-Utrilla A., Lopez-Prats F.A. (2015). Prognostic factors of health-related quality of life in patients after tibial plafond fracture. A pilot study. Injury.

[6] Jan Bartoniček, Mittlmeier Thomas, Rammelt Stefan (2012). Anatomie, Biomechanik und Pathomechanik des Pilon tibiale. Fuß & Sprunggelenk.

[7] Pollak A.N., McCarthy M.L., Bess R.S. (2003). Outcomes after treatment of high-energy tibial plafond fractures. J Bone Joint Surg Am.

[8] Lapidus L.J., Rosfors S., Ponzer S. (2007). Prolonged thromboprophylaxis with dalteparin after surgical treatment of achilles tendon rupture: a randomized, placebo-controlled study. J Orthop Trauma.

[9] Lapidus L.J., Ponzer S., Elvin A. (2007). Prolonged thromboprophylaxis with Dalteparin during immobilization after ankle fracture surgery: a randomized placebo-controlled, double-blind study. Acta Orthop.

[10] Nicholson M., Chan N., Bhagirath V. (2020). Prevention of Venous Thromboembolism in 2020 and beyond. J Clin Med.

[11] SooHoo N.F., Eagan M., Krenek L. (2011). Incidence and factors predicting pulmonary embolism and deep venous thrombosis following surgical treatment of ankle fractures. Foot Ankle Surg.

[12] Shibuya N., Frost C.H., Campbell J.D. (2012). Incidence of acute deep vein thrombosis and pulmonary embolism in foot and ankle trauma: analysis of the National Trauma Data Bank. J Foot Ankle Surg.

[13] Saad B.N., Yingling J.M., Liporace F.A. (2019). Pilon fractures: challenges and solutions. Orthop Res Rev.

[14] Zixuan L., Chen W., Li Y. (2020). Incidence of deep venous thrombosis (DVT) of the lower extremity in patients undergoing surgeries for ankle fractures. J Orthop Surg Res.

[15] Park J.S., Jang J.H., Park K.Y. (2018). High energy injury is a risk factor for preoperative venous thromboembolism in the patients with hip fractures: a prospective observational study. Injury.

[16] Basques B.A., Miller C.P., Golinvaux N.S. (2015). Risk factors for thromboembolic events after surgery for ankle fractures. Am J Orthop (Belle Mead NJ).

[17] Gonzalez Della Valle A., Shanaghan K.A., Nguyen J. (2020). Multimodal prophylaxis in patients with a history of venous thromboembolism undergoing primary elective hip arthroplasty. Bone Jt J.

[18] Yang Y., Li Z., Liang H. (2020). Association between metabolic syndrome and venous thromboembolism after total joint arthroplasty: a meta-analysis of cohort studies. J Orthop Surg Res.

[19] Sodhi N., Anis H.K., Acuña A.J. (2020). The effects of opioid use on thromboembolic complications, readmission rates, and 90-Day episode of care costs after total hip arthroplasty. J Arthroplast.

[20] Willenberg T., Schumacher A., Amann-Vesti B. (2010). Impact of obesity on venous hemodynamics of the lower limbs. J Vasc Surg.

[21] Blokhin I.O., Lentz S.R. (2013). Mechanisms of thrombosis in obesity. Curr Opin Hematol.

[22] Olson N.C., Cushman M., Lutsey P.L. (2014). Inflammation markers and incident venous thromboembolism: the REasons for Geographic and Racial Differences in Stroke (REGARDS) cohort. J Thromb Haemostasis.

[23] Ouchi N., Parker J.L., Lugus J.J. (2011). Adipokines in inflammation and metabolic disease. Nat Rev Immunol.

[24] Ogden C.L., Carroll M.D., Fryar C.D. (2015).

[25] Lindström S., Germain M., Crous-Bou M. (2018). Correction to: assessing the causal relationship between obesity and venous thromboembolism through a Mendelian Randomization study. Hum Genet.

[26] Apovian C.M. (2016). Obesity: definition, comorbidities, causes, and burden. Am J Manag Care.

[27] WHO (2000).

[28] Zhou B.F. (2002). Cooperative Meta-Analysis Group of the Working Group on Obesity in China. Predictive values of body mass index and waist circumference for risk factors of certain related diseases in Chinese adults--study on optimal cut-off points of body mass index and waist circumference in Chinese adults. Biomed Environ Sci.

[29] Brinkmann V., Reichard U., Goosmann C. (2004). Neutrophil extracellular traps kill bacteria. Science.

[30] Liu L., Zhang W., Su Y. (2021). The impact of neutrophil extracellular traps on deep venous thrombosis in patients with traumatic fractures. Clin Chim Acta.

[31] Gould T.J., Vu T.T., Swystun L.L. (2014). Neutrophil extracellular traps promote thrombin generation through platelet-dependent and platelet-independent mechanisms. Arterioscler Thromb Vasc Biol.

[32] Borissoff J.I., ten Cate H. (2011). From neutrophil extracellular traps release to thrombosis: an overshooting host-defense mechanism. J Thromb Haemostasis.

[33] Tripodi A., Ammollo C.T., Semeraro F. (2017). Hypercoagulability in patients with Cushing disease detected by thrombin generation assay is associated with increased levels of neutrophil extracellular trap-related factors. Endocrine.

[34] Zucoloto A.Z., Jenne C.N. (2019). Platelet-Neutrophil interplay: insights into Neutrophil extracellular trap (NET)-Driven coagulation in infection. Front Cardiovasc Med.

[35] Guo J., Yin Y., Jin L. (2019). Acute compartment syndrome: cause, diagnosis, and new viewpoint. Medicine (Baltim).

[36] Varela C.D., Vaughan T.K., Carr J.B. (1993). Fracture blisters: clinical and pathological aspects. J Orthop Trauma.

[37] Fox E.A., Kahn S.R. (2005). The relationship between inflammation and venous thrombosis. A systematic review of clinical studies. Thromb Haemost.

[38] Newby D.E., Wright R.A., Labinjoh C. (1999). Endothelial dysfunction, impaired endogenous fibrinolysis, and cigarette smoking: a mechanism for arterial thrombosis and myocardial infarction. Circulation.

[39] Haddix K.P., Clement R.C., Tennant J.N. (2018). Complications following operatively treated ankle fractures in Insulin- and non-insulin-dependent diabetic patients. Foot Ankle Spec.

[40] Werner B.C., Burrus M.T., Looney A.M. (2015). Obesity is associated with increased complications after operative management of end-stage ankle arthritis. Foot Ankle Int.

[41] Bian F.C., Cheng X.K., An Y.S. (2021). Preoperative risk factors for postoperative blood transfusion after hip fracture surgery: establishment of a nomogram. J Orthop Surg Res.

[42] Liu B., Pan J., Zong H. (2021). The risk factors and predictive nomogram of human albumin infusion during the perioperative period of posterior lumbar interbody fusion: a study based on 2015-2020 data from a local hospital. J Orthop Surg Res.

[43] Wang H., Fan T., Tang Z.R. (2021). Development and validation of a nomogram for prediction of the risk of positive hidden blood loss in the perioperative period of single-level thoracolumbar burst fracture. J Orthop Surg Res.

[44] Zhu J., Hu H., Deng X. (2021). Nomogram for predicting reoperation following internal fixation of nondisplaced femoral neck fractures in elderly patients. J Orthop Surg Res.

